# Household sanitation is associated with lower risk of bacterial and protozoal enteric infections, but not viral infections and diarrhoea, in a cohort study in a low‐income urban neighbourhood in Vellore, India

**DOI:** 10.1111/tmi.12915

**Published:** 2017-07-17

**Authors:** David Berendes, Juan Leon, Amy Kirby, Julie Clennon, Suraja Raj, Habib Yakubu, Katharine Robb, Arun Kartikeyan, Priya Hemavathy, Annai Gunasekaran, Sheela Roy, Ben Chirag Ghale, J. Senthil Kumar, Venkata Raghava Mohan, Gagandeep Kang, Christine Moe

**Affiliations:** ^1^ School of Civil and Environmental Engineering Georgia Institute of Technology Atlanta GA USA; ^2^ Center for Global Safe Water, Sanitation, and Hygiene Rollins School of Public Health Emory University Atlanta GA USA; ^3^ Hubert Department of Global Health Rollins School of Public Health Emory University Atlanta GA USA; ^4^ Department of Biostatistics Rollins School of Public Health Emory University Atlanta GA USA; ^5^ Wellcome Research Laboratory Christian Medical College Vellore India; ^6^ Department of Community Health Christian Medical College Vellore India

**Keywords:** sanitation, enteric infection, India, epidemiology, children, diarrhoea, Mots‐clés, assainissement, infection entérique, Inde, épidémiologie, enfants, diarrhée

## Abstract

**Objective:**

This study examined associations between household sanitation and enteric infection – including diarrhoeal‐specific outcomes – in children 0–2 years of age in a low‐income, dense urban neighbourhood.

**Methods:**

As part of the MAL‐ED study, 230 children in a low‐income, urban, Indian neighbourhood provided stool specimens at 14–17 scheduled time points and during diarrhoeal episodes in the first 2 years of life that were analysed for bacterial, parasitic (protozoa and helminths) and viral pathogens. From interviews with caregivers in 100 households, the relationship between the presence (and discharge) of household sanitation facilities and any, pathogen‐specific, and diarrhoea‐specific enteric infection was tested through mixed‐effects Poisson regression models.

**Results:**

Few study households (33%) reported having toilets, most of which (82%) discharged into open drains. Controlling for season and household socio‐economic status, the presence of a household toilet was associated with lower risks of enteric infection (RR: 0.91, 95% CI: 0.79–1.06), bacterial infection (RR: 0.87, 95% CI: 0.75–1.02) and protozoal infection (RR: 0.64, 95% CI: 0.39–1.04), although not statistically significant, but had no association with diarrhoea (RR: 1.00, 95% CI: 0.68–1.45) or viral infections (RR: 1.12, 95% CI: 0.79–1.60). Models also suggested that the relationship between household toilets discharging to drains and enteric infection risk may vary by season.

**Conclusions:**

The presence of a household toilet was associated with lower risk of bacterial and protozoal enteric infections, but not diarrhoea or viral infections, suggesting the health effects of sanitation may be more accurately estimated using outcome measures that account for aetiologic agents.

## Abstract (fr)

**Objectif:**

Cette étude a examiné les associations entre l'assainissement domestique et l'infection entérique, y compris les résultats spécifiques de la diarrhée, chez les enfants de 0 à 2 ans dans un quartier urbain dense à faible revenu.

**Méthodes:**

Dans le cadre de l’étude MAL‐ED, 230 enfants d'un quartier urbain indien à faible revenu ont fourni des échantillons de selles au cours de 14 à 17 points programmés et pendant les épisodes diarrhéiques au cours des deux premières années de vie, qui ont été analysés pour les pathogènes bactériens, viraux et les parasites (protozoaires et helminthes). A partir d'entretiens avec des soignants dans 100 ménages, la relation entre la présence (et le déversement) des installations d'assainissement domestiques et toute infection entérique spécifique au pathogène et à la diarrhée a été testée à l'aide de modèles de régression de Poisson à effets mixtes.

**Résultats:**

Peu de ménages dans l’étude (33%) ont déclaré avoir des toilettes, dont la plupart (82%) étaient déversées dans des égouts ouverts. En ajustant pour la saison et le statut socioéconomique du ménage, la présence de toilettes domestiques a été associée à de plus faibles risques d'infection entérique (RR: 0,91 ; IC95%: 0,79‐1,06), d'infection bactérienne (RR: 0,87 ; IC95%: 0,75‐1,02 ) et d'infection à protozoaires (RR: 0,64 ; IC95%: 0,39‐1,04), quoique non significatives statistiquement, mais il n'y avait aucune association avec la diarrhée (RR: 1,00 ; IC95%: 0,68‐1,45) ou les infections virales (RR: 1,12 ; IC95%: 0,79‐1,60). Les modèles ont également suggéré que la relation entre le déversement des toilettes des ménages dans les égouts et le risque d'infection entérique pouvait varier selon la saison.

**Conclusions:**

La présence de toilettes domestiques a été associée à un risque plus faible d'infections entériques bactériennes et à protozoaires, mais pas à la diarrhée ou aux infections virales, ce qui suggère que les effets sur la santé de l'assainissement peuvent être estimés plus précisément en utilisant des mesures de résultats qui tiennent compte des agents étiologiques.

## Abstract (es)

**Objetivo:**

Este estudio examinó las asociaciones entre el saneamiento doméstico y la infección entérica, incluyendo los resultados específicos de diarrea, en niños con 0‐2 años de edad de un barrio urbano denso y de bajos ingresos.

**Métodos:**

Como una parte del estudio MAL‐ED, se recogieron muestras de heces de 230 niños pertenecientes a un barrio urbano de bajos ingresos. Las muestras de heces se recogieron en 14‐17 momentos previamente programados y durante los episodios de diarrea de los primeros dos años de vida, y se analizaron en busca de patógenos bacterianos, parásitos (protozoos y helmintos) y virus. De las entrevistas con los cuidadores de 100 hogares, se miró la relación entre la presencia (y descarga) de sanitarios en los hogares y cualquier infección entérica específica de patógenos utilizando modelos de regresión de Poisson de efectos mixtos.

**Resultados:**

Pocos hogares de los que participaron en el estudio (33%) reportaron tener inodoros, la mayoría de los cuales (82%) descargaban a desagües abiertos. Al controlar para la estación y el estatus socioeconómico del hogar, la presencia de un sanitario estaba asociada con un menor riesgo de infección entérica (RR: 0.91, IC 95%: 0.79‐1.06), de infección bacteriana (RR: 0.87, IC 95%: 0.75‐1.02) e infección protozoaria (RR: 0.64, IC 95%: 0.39‐1.04) aunque no eran estadísticamente significativas, pero no existía asociación con la diarrea (RR: 1.00, 95% CI: 0.68‐1.45) o las infecciones víricas (RR: 1.12, IC 95%: 0.79‐1.60). Los modelos también sugieren que la relación entre los inodoros en hogares que descargan a alcantarillas y el riesgo de infección entérica puede variar según la estación.

**Conclusiones:**

La presencia de un sanitario doméstico estaba asociado con un menor riesgo de infecciones entéricas bacterianas y protozoarias, pero no con la diarrea o las infecciones víricas, sugiriendo que los efectos del saneamiento sobre la salud podrían ser determinados con mayor precisión utilizando medidas de resultados que tengan en cuenta los agentes etiológicos.

## Introduction

Despite an estimated 1.7 billion cases of diarrhoea annually, most of which are in children, the impact of enteric infections worldwide is underestimated due to high, undetected rates of asymptomatic infection 1, 2, 3. Even in the absence of diarrhoea, these infections are detrimental to child health, growth and cognition 4. Although these infections are thought to be driven by poor water, sanitation and hygiene (WASH), few studies have evaluated the impact of WASH on combined symptomatic and asymptomatic infections 5.

Current knowledge about the associations between WASH and enteric infections is limited by the lack of precision of common WASH outcome measures (e.g. self‐reported diarrhoea) and differences in study locations (e.g. rural *vs*. urban) 6, 7, 8, 9. For example, self‐reported diarrhoea reflects mixed aetiologic agents (e.g. including bacteria, intestinal parasites, and viruses) that individually vary greatly in their environmental persistence and infectious dose 3, 6, 7, 10, 11, 12. Further, self‐reported diarrhoea ignores asymptomatic infection and is subject to enumerator and respondent bias 13, 14, 15. In addition, while the WASH – and especially sanitation – research field has recently focused on rural settings in the context of the Millennium Development Goals, there is a need to understand the complex association between sanitation and health in urban areas, which are rapidly growing and already include over half of the world's population 16, 17, 18.

Urban environments can facilitate exposure to faecal contamination with aetiologic agents in both the household and public domains 17, 19, 20, 21, 22, 23, 24. When functioning, both household and public toilets, along with proper faecal sludge management (FSM), contain excreta from human contact throughout the sanitation chain at the household and neighbourhood levels 18, 25. However, these urban environments may easily become contaminated with faeces and aetiologic agents due to poor maintenance and cleanliness of the sanitation facility and poor FSM, which in turn pose risks to users and local residents 25, 26, 27, 28, 29, 30, 31, 32, 33, 34, 35.

The density and geography of an urban neighbourhood can affect FSM, and thus exposures to aetiologic agents in the public domain. Residents may either open defecate or use public toilets if the urban environment is too dense to construct new household toilets or find locations to empty the excreta from existing ones 33. The costs and logistics associated with either sewerage or toilet emptying and trucking make open drains a common fate for untreated excreta, posing exposure risks to children both when playing and through localised flooding 19, 20, 25, 36, 37, 38, 39, 40, 41, 42.

The goal of this study was to examine the associations between household sanitation and infection with different groups of enteric pathogens in a cohort of children under 2 years old in a low‐income, urban neighbourhood in Vellore, India. To better characterise these associations, the study accounted for the type of excreta containment associated with the household toilet, the type of aetiologic agent detected in stool and presence/absence of diarrhoea associated with the stool specimen. Examining the associations between household sanitation and enteric infection by type of aetiologic agent can inform our understanding of the role that the household environment plays in paediatric enteric infections in low‐income, urban settings.

## Methods

### Data sources

This study was a cohort study conducted in the Old Town neighbourhood of Vellore, India using two sources of data: (i) The Etiology, Risk Factors and Interactions of Enteric Infections and Malnutrition and Consequences for Child Health and Development Project (MAL‐ED study); and (ii) the SaniPath Exposure Assessment Tool 4, 43, 44. The MAL‐ED study in Vellore was conducted by the Christian Medical College and Hospital, Vellore (CMC) 43. The birth cohort was enrolled from March 2010 to February 2012, with data collection ending in February 2014. Exposure variables were characterised by household surveys as part of the SaniPath Tool deployment conducted by Emory University in February–March, 2014–after the completion of outcome assessment – in collaboration with CMC. A STROBE (strengthening the reporting of observational studies in epidemiology) checklist is provided in the Table S1.

### Study site

Annually, Vellore has a dry season (January–May), a southwest monsoon (June–September) and a northeast monsoon (October–December) 43. Old Town is a small, low‐income urban neighbourhood with high population density (approximately 42 000/km^2^), poor sanitation and high burden of enteric disease 35, 43. CMC has a longstanding relationship with the community, including mapping from previous studies 43.

### Ethical approval

Ethical approval for the MAL‐ED study in Vellore was obtained from the CMC Institutional Review Board (IRB) prior to subject recruitment 43. Approval was obtained from the Emory University IRB and the CMC IRB prior to the SaniPath Tool deployment. Informed consent was obtained onsite prior to survey administration.

### Stool specimen collection and testing

Stool specimens were collected from one study child per household as described in MAL‐ED study protocols 43, 45. Specimens were collected monthly over the child's first year of life, and then every 2–3 months over the next year (‘routine stool’). Caregivers also submitted specimens at each diarrhoeal event during the study period (‘diarrhoeal stool’). All specimens were tested for bacteria, protozoa, helminths and viruses (organism list available in the Table S1) by culture, microscopy, immunoassay and PCR as described previously 2, 10. Pathogen detection was then aggregated by group of aetiologic agents (e.g. bacterial agents, viral agents) prior to modelling.

### Household survey data collection: SaniPath Tool

Household selection has been previously described in detail 35. Briefly, 100 households were surveyed (as prespecified in the SaniPath tool), 25 of which were selected based on the results of a hygiene survey completed prior to SaniPath Tool deployment to ensure diversity of household hygiene practices 46. The other 75 households were chosen randomly in the MAL‐ED study area 43.

Household surveys assessed demographics and sanitation, defecation practices of household members and reported water treatment. The target respondent was the female head of household. GPS points were collected at the time of household survey.

### Analyses

Analyses were conducted in R version 3.1.2 (R Foundation for Statistical Computing, Vienna, Austria) using standard packages and the ‘lme4’ package for mixed‐effects models 47. Enteric infection was defined as the presence of one or more enteric pathogens detected in an asymptomatic or symptomatic stool specimen collected from children in study households during the study period. Similarly, bacterial infection, viral infection, protozoal infection or helminth infections were defined, respectively, as the presence of one or more of these types of pathogens detected in an asymptomatic or symptomatic stool specimen. Diarrhoea was defined from caregiver‐submitted diarrhoeal stool specimens (as compared to asymptomatic, ‘routine’ stool specimens collected). Missing outcome data for a given time point of stool collection was excluded. Households lost to follow‐up were not included in the initial SaniPath Tool household sampling frame.

Mixed‐effects Poisson regression models with a random effect for the child sampled were used to estimate associations between household sanitation and the risk of enteric infection or diarrhoea. Parasitic aetiologic agents were modelled individually as soil‐transmitted helminths or protozoa based on the potential for differential associations with environmental conditions 12. Household sanitation was examined by type using questions from household surveys (collected at the end of the MAL‐ED follow‐up period) about presence of a toilet and fate of the excreta (e.g. toilet leads to drain, excreta is contained in a tank onsite) and stratified by season (defined by month) to assess the association between the quality of excreta containment and risk of enteric infection. No missing data were observed in the exposure assessment. Unadjusted and adjusted models were assessed. Multivariable models were prespecified to control for season of specimen collection (dry, southwest (SW) monsoon, or northeast (NE) monsoon) and household socio‐economic status (SES), defined by the inclusion of the household asset index, household income and mother's highest education categories (0–8 for each) acquired from a location‐specific socio‐economic metric for the MAL‐ED study 48. To avoid multicollinearity with the primary exposure, water and sanitation measures were excluded from the metric for this study. This information was collected at three to four time points during the follow‐up period. Scores for household assets, income and mother's education were assigned to stool specimens based on date of collection (i.e. all specimens collected between the first and second household socio‐economic assessment were assigned the values of the first assessment, while all collected between the second and third assessment were assigned the values of the second assessment). Any specimens collected before a household socio‐economic assessment was conducted were assigned values from the first assessment. An *α* of 0.05 was used for all tests of significance.

Given the reversal in timing of exposure and outcome assessment, a sensitivity analysis of the presence of household sanitation, approximated using data collected at three to four time points during the follow‐up period for a ‘use of sanitation facility’ question as part of the socio‐economic metric 48, was analysed to assess the validity of static assumptions.

## Results

### Household sanitation and defecation behaviours in the study neighbourhood

Characteristics of the household and neighbourhood environments and child exposure behaviours were quantified through surveys with the adult caregiver (Table 1). Overall, 100 households (100 children) were assessed for exposures (no missing values) at the end of the study period (February 2014). Few households (33%) reported having toilets, and most household toilets (82%) discharged directly into an open drain. Only three households reported having a toilet that discharged into a ‘septic tank’ containing excreta onsite. Open drains were ubiquitous near households, and many respondents (58%) reported that the drains in front of their household would flood (data not shown). Open defecation was common across all age groups. Notably, all households reported either using their household toilet or open defecating, and not using public toilets, as their primary location of defecation. However, approximately half of all households reported using public toilets at least once a month, although only 13% reported frequent use (>10 times per month). Almost one‐third of households reported regularly treating their drinking water.

**Table 1:** Reported household/neighbourhood conditions and exposure behaviours^a^

Household conditions	Count (%) or Mean (SD)
Demographics	
5–12 year old present	62 (62)
Average number of people per household	6.4 (2)
Sanitation	
Household toilet	33 (33)
With poor FSM^b^ (discharging to drain)	27 (82)
With good FSM^b^ (excreta contained onsite)	3 (9)
Other/Don't know	3 (9)
Open drain in front of household	96 (96)
Open defecation	
<5 year olds	80 (80)
5–12 year olds	45 (79)
Adult	68 (68)
Public toilet use	
Any	46 (46)
>10 times per month	13 (13)
Reported water treatment	
Drinking water treatment at the household	32 (32)

a Data from household survey (*n* = 100 households).

b Faecal sludge management (describing the containment of excreta along the entire sanitation chain, from toilet to treatment 25, 78).

### Distribution of pathogenic organisms in children's stool

The presence of enteropathogens in stool specimens was compared by type of stool specimen (routine (asymptomatic stool collected at regular intervals) *vs*. diarrhoeal) to characterise the infection burden, and by study population (subset of SaniPath study households *vs*. all MAL‐ED study households) to compare the representativeness of the SaniPath study households to the overall MAL‐ED study. A total of 3754 stool specimens were collected from children during the MAL‐ED study, 1650 of which were collected from the 100 children in SaniPath study households. Approximately 68% of routine and 79% of diarrhoeal stool specimens were positive for enteric infection (at least one pathogen detected, Table 2). Bacterial pathogens were most frequently detected in stool specimens of both types. Detection of enteropathogens – both generally and by type – in stool from children in SaniPath households did not differ significantly from that of children in all the MAL‐ED study households, with the exception of detection of helminths, which was significantly higher in children in all MAL‐ED study households compared with SaniPath study households. Because of the low prevalence of helminth infection detected in children in SaniPath households, however, helminth infections were excluded from further modelling.

**Table 2:** Detection of pathogens in children's stool in SaniPath and all MAL‐ED households from 2010 to 2014

Single infections	Per cent of child's stool specimens collected that were positive^a^					
	Stool collected from children in SaniPath households (*n* = 100 children)^b^			Stool from children in all MAL‐ED study households (*n* = 230 children)^c^		
	Routine collection^d^	Diarrhoeal^e^	All stool collected	Routine collection^d^	Diarrhoeal^e^	All stool collected
Any pathogen^f^	67.2	82.6	69.2	67.6	79.4	69.2
Bacterial infection	60.6	64.5	60.9	59.9	61.0	59.8
Parasitic infection^g^	16.1	18.3	16.3	17.9	21.3	18.3
Helminth infection	0.6	0.0	0.5	2.9	3.9	2.9
Protozoal infection	15.8	18.3	16.1	16.0	18.0	16.4
Viral infection	7.9	39.4	12.5	8.6	35.9	12.8
Combined infections						
Viral + bacterial	5.6	26.4	8.6	6.4	23.7	9.0
Viral + parasite	1.4	6.2	2.2	1.8	6.6	2.6
Bacterial + parasite	11.6	12.2	11.7	12.2	13.4	12.3
Bacterial, viral, and parasite	1.2	4.8	1.8	1.6	4.8	2.2

a Calculated as the average, by child, of the proportion of stool specimens that were positive for a given pathogen.

b SaniPath households consisted of a subset of all MAL‐ED study households that were surveyed for demographics, exposure behaviours, and household and local neighbourhood conditions. A total of 1650 specimens were collected from children in these households during the study period. Only significant differences in helminth detection were observed in stool from children in SaniPath households compared to children in all MAL‐ED study households, detection of all other pathogens were similar in both groups.

c *N* = 3754 stool specimens collected.

d Routine stool was collected monthly over the first year of follow‐up, then every 2–3 months during the second year of follow‐up.

e Diarrhoeal stool collected whenever a child had an episode of diarrhoea, as reported by the caregiver.

f A full list of pathogens tested in stool specimens is available in the supplemental information and Houpt *et al*. 2014

g Includes protozoa and soil‐transmitted helminths.

### Pathogens associated with diarrhoeal stool

The associations between pathogen presence and the risk of diarrhoeal stool were evaluated using mixed‐effects Poisson regression (data not shown). Virus presence was associated with significantly higher risk of diarrhoeal stool (risk ratio (RR) for viral detection: 4.37, 95% confidence interval (CI): 3.39–5.63). Bacterial presence (RR: 1.17, 95% CI: 0.90–1.53) and protozoal presence (RR: 0.83, 95% CI: 0.61–1.14) were not significantly associated with risk of diarrhoeal stool.

### Seasonality of enteric infection risk

Seasonality of enteric infection risk was evaluated by mixed‐effects Poisson regression for all stool specimens in the SaniPath study households. Overall, the risk of enteric infection (defined as presence of any pathogen in stool) did not vary significantly by season (data not shown). Viral infections were 40% less likely during the northeast monsoon (October–December) than during the dry season (January–May, RR: 0.60, 95% CI: 0.40–0.90, *P* = 0.01). Risk of bacterial infection, protozoal infection and diarrhoea did not differ significantly by season (data not shown).

### Unadjusted associations between household sanitation, socioeconomic status, and enteric infection or diarrhoea

Unadjusted associations between household sanitation, SES measures, and risk of enteric infection or diarrhoea were measured in mixed‐effects Poisson regression models (Table 3). Presence of a household toilet was associated with 8% reduced risk of enteric infection overall (RR: 0.92, 95% CI: 0.81–1.04), a 12% reduced risk of bacterial infections (RR: 0.88, 95% CI: 0.77–1.00) and a 31% reduced risk of protozoal infection (RR: 0.69, 95% CI: 0.45–1.05), although none were statistically significant. Risk of viral infections and risk of diarrhoea were not associated with household toilet presence. Risk of enteric infections, both generally and organism‐specific, as well as risk of diarrhoea, was not significantly associated with household assets or mother's education, with the exception of risk of protozoal infection, which decreased by 11% per category of mother's education. Notably, risk of viral infection was associated with an 8% increase per increasing income category, although was not significant.

**Table 3:** Unadjusted relationships between household sanitation, socioeconomic status, and enteric infection or diarrhoea measured from stool collected from children in SaniPath households, 2010–2014†

Poisson model main effect	Enteric infection (any pathogen)‡ RR (95% CI)	Bacterial infection§ RR (95% CI)	Protozoal infection¶ RR (95% CI)	Viral infection∥ RR (95% CI)	Diarrhoea†† RR (95% CI)
Household toilet	0.92 (0.81, 1.04)	0.88 (0.77, 1.00)	0.69 (0.45, 1.05)	1.10 (0.81, 1.49)	1.15 (0.83, 1.60)
Household toilet to drain	0.92 (0.81, 1.05)	0.89 (0.77, 1.03)	0.77 (0.49, 1.21)	0.91 (0.65, 1.28)	1.17 (0.83, 1.65)
Asset index (0–8)‡‡	0.99 (0.97, 1.02)	0.99 (0.96, 1.02)	1.00 (0.91, 1.09)	1.02 (0.95, 1.09)	1.07 (0.99, 1.15)
Income (0–8)‡‡	1.02 (0.98, 1.06)	1.01 (0.97, 1.04)	1.04 (0.95, 1.15)	1.08 (1.00, 1.18)	1.03 (0.94, 1.12)
Mother's education (0–8)‡‡	0.99 (0.96, 1.02)	0.99 (0.95, 1.02)	0.89 (0.80, 0.99)*	1.03 (0.94, 1.12)	1.03 (0.94, 1.12)

†Helminth infections were not included in unadjusted and adjusted (Table 4) analyses due to low numbers of positive detections (*N* = 8/1650).

‡*N* = 1149/1650.

§*N* = 1009/1650.

¶*N* = 270/1650.

∥*N* = 215/1650.

††*N* = 264/1650.

‡‡Site‐specific categories estimated for household SES indices using methods described previously (48).

*P < 0.05; **P < 0.01.

### Multivariable associations between household sanitation and enteric infection or diarrhoea

Associations between household sanitation and risk of enteric infection and diarrhoea were compared, controlling for season, household SES (via asset index, household income and mother's education, Table 4). Relationships between household toilet FSM and season were also evaluated, although these were limited to household toilets emptying to drains because too few (*n* = 3) households had toilets that contained waste onsite. Although not significant, risk of enteric infection (infection with any pathogen) was 9% lower in children in households with toilets compared to those without. Risks of bacterial and protozoal infections for children in households with toilets were 13% and 36% lower (bacterial infection RR: 0.87, 95% CI: 0.75–1.02; protozoal infection RR: 0.64, 95% CI: 0.39–1.04), respectively, than for children in households without toilets, although these relationships were not significant. No association was observed between the presence of household toilets and risk of viral infection (RR: 1.12, 95% CI: 0.79–1.60) or risk of diarrhoea (RR: 1.00, 95% CI: 0.68–1.45). Although not significant, the associations between household toilets that emptied to open drains and enteric infection risk suggested seasonal variation: the presence of a toilet was associated with a 7% decreased risk overall and a 14% decreased risk during the dry season; however, during the northeast monsoon season – the heaviest period of rain, the presence of a toilet did not suggest any protective effects. Similar trends were also observed for risks of bacterial and protozoal infections.

**Table 4:** Multivariable relationships between household sanitation, season, and enteric infection or diarrhoea measured from stool collected from children in SaniPath households, 2010–2014^c^

Poisson model main effect	Enteric infection (any pathogen) RR (95% CI)	Bacterial infection RR (95% CI)	Protozoal infection RR (95% CI)	Viral infection RR (95% CI)	Diarrhoea RR (95% CI)
Household toilet^a^	0.91 (0.79, 1.06)	0.87 (0.75, 1.02)	0.64 (0.39, 1.04)	1.12 (0.79, 1.60)	1.00 (0.68, 1.45)
Household toilet to drain^a^	0.93 (0.80, 1.07)	0.90 (0.77, 1.05)	0.74 (0.45, 1.21)	0.89 (0.62, 1.27)	1.05 (0.73, 1.52)
Dry season^b^	0.86 (0.69, 1.08)	0.86 (0.67, 1.09)	0.56 (0.28, 1.12)	0.87 (0.50, 1.54)	1.02 (0.64, 1.63)
SW monsoon^b^	0.92 (0.72, 1.16)	0.83 (0.64, 1.07)	0.88 (0.49, 1.57)	1.19 (0.73, 1.92)	1.26 (0.81, 1.97)
NE monsoon^b^	1.07 (0.79, 1.44)	1.11 (0.81, 1.53)	0.96 (0.52, 1.77)	0.40 (0.14, 1.17)	0.70 (0.33, 1.48)

a Models adjusted for monsoon seasons (relative to dry season), asset index, income, and mother's education.

b Models adjusted for asset index, income, and mother's education.

c Season‐specific analyses were not possible for helminth infections due to low numbers of positive detections (*n* = 8/1650).

### Temporal assessment of misclassification of exposure

Sensitivity analysis of toilet presence over time revealed that some misclassification may have existed in assignment of the primary exposure over the study period. Using temporal assignment of socio‐economic metrics similar to those for household assets, income or maternal education as described in the methods, up to 198 (12%) of stool specimens collected may have been misclassified (i.e. households reported having a toilet at SaniPath exposure assessment after MAL‐ED follow‐up, but did not report using it at time of socio‐economic assessment or vice‐versa).

## Discussion

The goal of this study was to evaluate the associations between household sanitation and paediatric enteric infections and diarrhoea in a low‐income, urban setting, focusing on associations with specific groups of aetiologic agents and diarrhoeal episodes. Household sanitation was generally associated with a lower risk of enteric infection, including lower risk of bacterial and protozoal enteric infections specifically, but was not associated with the risk of viral infections. Household sanitation was not associated with the risk diarrhoea in these children, suggesting that relationships between household toilets and health outcomes may be more appropriately evaluated with a specific focus on the type of enteric infection, rather than more nonspecific symptoms like diarrhoea. Although the presence of a household toilet was largely protective, data from households with toilets that discharged to open drains suggested a potential seasonal variation in the effectiveness of this specific type of toilet.

This study is unique in testing associations between urban household sanitation and enteric infection in a paediatric cohort that captured most enteric infections and diarrhoea during the first 2 years of life and comparing risk factors by type of infection. While previous studies have explored risk factors for diarrhoeal disease in urban contexts, few have included laboratory‐confirmed enteric infections or examined infections by specific groups of pathogens in the analysis 24, 37, 49, 50, 51, 52, 53, 54, 55, 56, 57, 58, 59. Urban studies of environmental transmission of infection with specific types of pathogens have primarily used quantitative microbial risk assessment 19, 20, 23.

Although the study children were too young to use a toilet for much of the study period, children's contact with older family members may have connected them to faecal contamination, emphasising the importance of household toilet use by all family members in lieu of open defecation. Beyond contaminating the local environment, open defecation by older family members increased their own direct and indirect (e.g. through flies) contact with faecal contamination compared with household toilet use 60. For example, in a similar study area in Vellore, households with a toilet had significantly lower density of flies and incidence of diarrhoeal disease in young children, compared to those without a toilet 46. In other urban settings, the presence of an improved household toilet was associated with lower levels of faecal contamination on hands 27.

We observed associations between household toilet presence and lower risk of bacterial and protozoal infections, but not viral infections, suggesting that use of toilets (and therefore sequestration of large volumes of faeces from human contact) may have been more effective in preventing bacterial and protozoal infections than infections with viruses. These findings may be due to differences between bacteria or protozoa and viruses in their transmission pathways and infectious doses, which in turn influenced their relationship with household sanitation. Compared to bacterial and protozoal infections, viral infections have more direct person‐to‐person contact and transmission via fomites because of their lower infectious doses and high titre shedding 61, 62, 63, 64. Viruses generally have median infectious doses of under 100 particles, much lower than the 10^6^–10^8^ required by many enteric bacterial pathogens 12, 62.

There were no observed differences in risk of diarrhoea in children with or without household toilets, suggesting that any changes in exposure to faecal contamination for young children associated with household toilets were not associated with symptomatic infections specifically. Recent reanalysis of data from the Global Enteric Multicenter Study (GEMS) using quantitative molecular methods has shown strong quantity‐dependent associations between detection levels in stool and diarrhoea for only a handful of enteric organisms, specifically *Shigella* spp. and heat‐stable enterotoxin‐producing *E. coli* (ST‐ETEC) 65. Most other enteric organisms, including many bacteria, were at most moderately associated with diarrhoea, suggesting that human health responses to environmental conditions may be more accurately detected by measurement of enteric organisms in stool 65, 66. Further, recent evidence from multiple studies suggests that controlling enteric infection, even at subclinical levels, is important for longer‐term health outcomes 67, 68, 69. Considering the large proportion of diarrhoea associated with viral pathogens in this study, these findings provide evidence to support future sanitation studies excluding viral pathogens or diarrhoeal episodes from primary outcome measures, and instead focusing on enteric infections by bacterial and protozoal agents, which may be more likely to be impacted by interventions to reduce exposure to faecal contamination in the environment 66.

Although not significant, we observed evidence that the relationship between household toilets leading to open drains and risk of enteric infection may vary by season. While further research is needed to substantiate this observation, it may reflect the poor containment of faeces from these toilets. Owning and using toilets that discharged to open drains may have been of limited benefit during periods of heavy monsoon or flooding because of the high levels of human‐specific faecal contamination discharged into the immediate environment 35. Previous cross‐sectional evidence from this population has shown that children in households with toilets that discharged to open drains had the highest prevalence of enteric infection, even compared to those in households without toilets, suggesting that contamination of the immediate environment may have reduced the health benefits associated with toilet ownership 35.

This study has some notable strengths and limitations in outcome and exposure measurements. Measurement of enteric pathogens in stool specimens collected regularly over the first 2 years of life provided a rich dataset that allowed sensitive assessment of the relationships between household sanitation and multiple health outcomes, including symptomatic and asymptomatic enteric infections 2, 3, 4, 6, 7. Given that pathogens can continue to be shed in stool for weeks or months after symptoms resolve 63, 64, separation of individual infection events in the dataset was difficult; however, adjustment for lack of independence of longitudinal stool collected from the same child removed some bias amongst the correlated data. Further, although data collection was designed to provide a comprehensive assessment of enteric infections ‐and diarrhoea specifically – during the follow‐up period 4, the passive surveillance with regard to diarrhoeal stool may have missed some diarrhoeal incidence.

Static assumptions were necessary to characterise household sanitation practices because household surveys were conducted at the end of the MAL‐ED study period. Sensitivity analyses suggested low levels of temporal misclassification of household sanitation, although these may have been sufficient to affect study power in determining statistical significance for many estimates. Exact determination of misclassification was hindered slightly by differences in the question administered to the respondent (presence/absence of household toilet in exposure assessment *vs*. use of sanitation facility in socio‐economic assessments). Assessment of household SES was robust, given that household assets, household income, and mother's education were assessed at three to four time points during the follow‐up period and classified into MAL‐ED site‐specific categories 48.

The results of this study are important for mitigation of paediatric enteric infections in under 2‐year‐olds, especially in dense, urban settings. Children in this study were enrolled at birth and selected at random for outcome follow‐up, with robust, comprehensive measures of enteric infections over the entire 2‐year period, when children may be most susceptible to growth‐faltering due to repeat enteric infections 70.

Mitigation of paediatric enteric infection by household sanitation may have important implications for malnutrition, growth and cognitive development, independent of diarrhoea 67, 71. While the mechanisms of association have been proposed and examined in cross‐sectional studies, there are several ongoing and recently completed randomised and controlled before‐and‐after studies examining these impacts that will improve understanding of the environmental and biological mechanisms involved 66, 72, 73, 74. Given the reliability and relative feasibility of molecular methods, including parallel and multiplex assays like the TaqMan Array Card and Luminex xTAG^®^ system, future sanitation studies should consider measures of enteric organisms present in stool to better understand how changes to the immediate household environment cause changes in faecal exposures and infection 65, 75, 76. Further, following the Sustainable Development Goals, measurement of the containment of faeces should be examined along the entire sanitation chain, and not only at the household 77.

In this setting, the presence of a household toilet was associated with lower risk of paediatric bacterial and protozoal infections, but not viral infections or diarrhoea. Further evidence suggested that these associations may vary by season, likely due to toilets discharging excreta directly into open drains and subsequent spread of faecal‐contaminated floodwaters during the heaviest rainy season. This study contributes to growing evidence around the importance of measuring enteric infections, and not solely diarrhoea, as the primary health outcome in studies of household sanitation 2, 67, 69, 71.

## Supporting information


**Table S1**. STROBE Statement—checklist of items that should be included in reports of observational studies.
