# Supplementary material for: Household sanitation is associated with lower risk of bacterial and protozoal enteric infections, but not viral infections and diarrhoea, in a cohort study in a low‐income urban neighbourhood in Vellore, India
Source: Trop Med Int Health. 2017 Jul 17;22(9):1119–29. doi: 10.1111/tmi.12915 (PMC5601219; doi:10.1111/tmi.12915)
Supplement: Supplementary file 1 — Table S1. STROBE Statement—checklist of items that should be included in reports of observational studies. [file TMI-22-1119-s001.docx]

Supplemental information (SI):

**Table S1** STROBE Statement—checklist of items that should be included in reports of observational studies

|  | Item No. | Recommendation | Page  No. | Relevant text from manuscript |
| --- | --- | --- | --- | --- |
| **Title and abstract** | 1 | (*a*) Indicate the study’s design with a commonly used term in the title or the abstract | 1 | Lines 1-3 |
|  |  | (*b*) Provide in the abstract an informative and balanced summary of what was done and what was found | 1-2 | Lines 22-43 |
| Introduction | | | |  |
| Background/rationale | 2 | Explain the scientific background and rationale for the investigation being reported | 3-4 | Lines 45-75 |
| Objectives | 3 | State specific objectives, including any prespecified hypotheses | 4 | Lines 76-83 |
| Methods | | | |  |
| Study design | 4 | Present key elements of study design early in the paper | 5 | Lines 86, 90-94 |
| Setting | 5 | Describe the setting, locations, and relevant dates, including periods of recruitment, exposure, follow-up, and data collection | 5 | Lines 86-100 |
| Participants | 6 | (*a*) *Cohort study*—Give the eligibility criteria, and the sources and methods of selection of participants. Describe methods of follow-up  *Case-control study*—Give the eligibility criteria, and the sources and methods of case ascertainment and control selection. Give the rationale for the choice of cases and controls  *Cross-sectional study*—Give the eligibility criteria, and the sources and methods of selection of participants | 6 | Lines 107-114, 116-123 |
|  |  | (*b*) *Cohort study*—For matched studies, give matching criteria and number of exposed and unexposed  *Case-control study*—For matched studies, give matching criteria and the number of controls per case | N/A | N/A |
| Variables | 7 | Clearly define all outcomes, exposures, predictors, potential confounders, and effect modifiers. Give diagnostic criteria, if applicable | 6-8 | Lines 127-133, 140-157 |
| Data sources/ measurement | 8* | For each variable of interest, give sources of data and details of methods of assessment (measurement). Describe comparability of assessment methods if there is more than one group | *6-8* | *Lines 127-133, 140-156* |
| Bias | 9 | Describe any efforts to address potential sources of bias | 7 | Lines 158-161 |
| Study size | 10 | Explain how the study size was arrived at | 6 | Line 116-119 |

Continued on next page

| Quantitative variables | 11 | Explain how quantitative variables were handled in the analyses. If applicable, describe which groupings were chosen and why | N/A |  |
| --- | --- | --- | --- | --- |
| Statistical methods | 12 | (*a*) Describe all statistical methods, including those used to control for confounding | 6-8 | 125-157 |
|  |  | (*b*) Describe any methods used to examine subgroups and interactions | 6-8 | 125-157 |
|  |  | (*c*) Explain how missing data were addressed | 7 | Line 133-135, 144 |
|  |  | (*d*) *Cohort study*—If applicable, explain how loss to follow-up was addressed  *Case-control study*—If applicable, explain how matching of cases and controls was addressed  *Cross-sectional study*—If applicable, describe analytical methods taking account of sampling strategy | 7 | Line 134-35 |
|  |  | (*e*) Describe any sensitivity analyses | 8 | Lines 158-161 |
| Results | | | | |
| Participants | 13* | (a) Report numbers of individuals at each stage of study—eg numbers potentially eligible, examined for eligibility, confirmed eligible, included in the study, completing follow-up, and analysed | 8, 9 | 165-6, 182-4 |
|  |  | (b) Give reasons for non-participation at each stage | N/A |  |
|  |  | (c) Consider use of a flow diagram | N/A |  |
| Descriptive data | 14* | (a) Give characteristics of study participants (eg demographic, clinical, social) and information on exposures and potential confounders | 8-9 | 164-176 |
|  |  | (b) Indicate number of participants with missing data for each variable of interest | 8 | 166 |
|  |  | (c) *Cohort study*—Summarise follow-up time (eg, average and total amount) | 8 | 196-7 |
| Outcome data | 15* | *Cohort study*—Report numbers of outcome events or summary measures over time | 9  26 | 178-191  Table 3 footnotes |
|  |  | *Case-control study—*Report numbers in each exposure category, or summary measures of exposure |  |  |
|  |  | *Cross-sectional study—*Report numbers of outcome events or summary measures |  |  |
| Main results | 16 | (*a*) Give unadjusted estimates and, if applicable, confounder-adjusted estimates and their precision (eg, 95% confidence interval). Make clear which confounders were adjusted for and why they were included | 9-11 | 207-237 |
|  |  | (*b*) Report category boundaries when continuous variables were categorized | N/A | N/A |
|  |  | (*c*) If relevant, consider translating estimates of relative risk into absolute risk for a meaningful time period | N/A | N/A |

Continued on next page

| Other analyses | 17 | Report other analyses done—eg analyses of subgroups and interactions, and sensitivity analyses | 11-12 | 232-237, 238-244 |
| --- | --- | --- | --- | --- |
| Discussion | | | | |
| Key results | 18 | Summarise key results with reference to study objectives | 12-14 | 245-309 |
| Limitations | 19 | Discuss limitations of the study, taking into account sources of potential bias or imprecision. Discuss both direction and magnitude of any potential bias | 14-15 | 310-330 |
| Interpretation | 20 | Give a cautious overall interpretation of results considering objectives, limitations, multiplicity of analyses, results from similar studies, and other relevant evidence | 16 | 348-354 |
| Generalisability | 21 | Discuss the generalisability (external validity) of the study results | 15-16 | 331-335 |
| Other information | |  | | |
| Funding | 22 | Give the source of funding and the role of the funders for the present study and, if applicable, for the original study on which the present article is based | 17 | 356-363 |

*Give information separately for cases and controls in case-control studies and, if applicable, for exposed and unexposed groups in cohort and cross-sectional studies.

**Note:** An Explanation and Elaboration article discusses each checklist item and gives methodological background and published examples of transparent reporting. The STROBE checklist is best used in conjunction with this article (freely available on the Web sites of PLoS Medicine at http://www.plosmedicine.org/, Annals of Internal Medicine at http://www.annals.org/, and Epidemiology at http://www.epidem.com/). Information on the STROBE Initiative is available at www.strobe-statement.org.

*Organisms tested in stool specimens:*

All stool specimens collected from children in the Old Town neighborhood were tested for the following bacteria: *Salmonella*, *Shigella*, *Vibrio*, *Yersinia*, *Aeromonas*, *Plesiomonas*, diarrheagenic *E. coli* (Shiga toxin-producing *E. coli*, enterotoxigenic *E. coli*, enteropathogenic *E. coli*, enteroinvasive *E. coli*, and enteroaggregative *E. coli*), and *Campylobacter* spp. Protozoa tested included *Balantidium coli*, *Cryptosporidium*, *Chilomastix mesnili*, *Cyclospora*, *Entamoeba histolytica*, *Giardia lamblia*, *Endolimax nana*, *Iodamoeba butschlii*, *Isospora*. Helminths included *Hymenolepis nana*, *Strongyloides stercoralis*, *Ascaris lumbricoides*, *Taenia*, *Trichuris trichiura*, *Schistosoma*, *Enterobius vermicularis*, *Hymenolepis diminuta*, and hookworm species. Finally, viruses included genogroup I and II norovirus, astrovirus, rotavirus, and adenovirus (2,10).
